# Toward a better understanding of chronic hepatitis B virus infection

**DOI:** 10.1172/JCI185568

**Published:** 2024-10-01

**Authors:** Barbara Rehermann

**Affiliations:** Immunology Section, Liver Diseases Branch, National Institute of Diabetes and Digestive and Kidney Diseases, National Institutes of Health, Department of Health & Human Services, Bethesda, Maryland, USA.

An intriguing phenomenon of HBV infection is the marked distinction of disease presentation and outcome depending on the age of the individual at infection (Figure 1). HBV infection during adulthood results in an acute, often severe, phase of liver inflammation, which is attributed to a strong adaptive immune response. The disease is self-limited in the vast majority (95%) of cases and results in life-long cellular and humoral immunity. The characteristics of this successful, natural immune response are well understood, biomarkers for protective immunity have been established, and protective vaccines are widely available and effective. In contrast, HBV infection in early life results in persistent, life-long infection, currently affecting more than 254 million people worldwide. Despite exceedingly high HBV levels in the serum, the infection typically does not result in clinical symptoms in the first decades. Liver inflammation, cirrhosis, and cancer generally occur long after the infected person has reached reproductive age, which points to coevolution of virus and host. Indeed, HBV sequences have been found in 400-year-old preserved mummies and in 3,000- to 7,000-year-old fossils from the Bronze and Neolithic ages (reviewed in ref. 1). HBV’s family of *Hepadnaviridae* has been tracked into an even more distant past and is thought to have diverged from nonenveloped DNA viruses in fish 400 million years ago (2). This long process of coadaptation and coevolution of HBV with its host likely explains why it has proven so difficult to cure chronic infection.

This Viewpoint, in honor of the 100th year anniversary of the *Journal of Clinical Investigation*, highlights research papers that have been published in this journal and identifies open questions for further translational immunological research on chronic hepatitis B.

## HBV’s complex life cycle

HBV is an enveloped, partially double-stranded DNA virus with an unusual life cycle. After entering hepatocytes via binding to the bile acid transporter (sodium taurocholate cotransporting polypeptide, NTCP), the nucleocapsid releases its cargo, the 3.2 kB relaxed circular DNA (rcDNA). It is transported to the hepatocyte nucleus, where it is repaired by host enzymes to covalently closed circular DNA (cccDNA). The cccDNA forms a stable, nonintegrated minichromosome and serves as transcriptional template for all viral mRNAs, which are then transported into the cytoplasm of the hepatocytes, and structural proteins and viral polymerase are synthesized (reviewed in ref. 3). HBV replicates within nucleocapsids in the cytoplasm by reverse transcription of pregenomic RNA into new rcDNA. The mature capsids transport rcDNA back to the nucleus to replenish the cccDNA or are enveloped and secreted from the cell. Infected cells also release noninfectious subviral filaments and particles in quantities that exceed those of infectious viral particles by 1,000- to 10,000-fold. Integration of HBV sequences into the host genome results in continuing production of hepatitis B surface antigen (HBsAg) during antiviral treatment (4).

## Immune responses in acute, self-limited hepatitis B

Characteristics of acute HBV infection are a delayed increase in serum HBV DNA levels; a surprising lack of innate immune responses, which has been attributed to HBV replication occurring within its nucleocapsid; and a clear sequence of adaptive immune responses with antibodies against HBV core antigen appearing first, followed by seroconversion from HBeAg^+^ to anti-HBe^+^ and from HBsAg^+^ to anti-hepatitis B surface^+^ (anti-HBs^+^). This antibody response is accompanied by a strong, multispecific CD4^+^ and CD8^+^ T cell response. Adults who recover from acute HBV infection maintain anti-HBs antibodies and memory T cell responses for decades after recovery, and this is essential to control small amounts of HBV that originate from persistence of cccDNA (3, 5). Accordingly, recovered patients are at risk of HBV reactivation if chemotherapy-induced immunosuppression occurs without concomitant antiviral therapy. Studies in chimpanzees (the sole animal model supporting the entire HBV life cycle) demonstrated the relative roles of cytokine-mediated versus cytolytic CD8^+^ T cell responses in HBV controls (reviewed in ref. 6). These studies also demonstrated that the strength of the CD8^+^ T cell response depends on CD4^+^ T cells and that the latter is reduced and delayed if the size of the viral inoculum is small (7).

## Immune responses in chronic hepatitis B

Baruch Blumberg’s 1965 discovery of HBsAg as a specific virological marker led to the realization that HBV causes a chronic form of hepatitis of worldwide prevalence (reviewed in ref. 8). Notably, despite decades of infection, a small percentage (~1% annually) of all patients spontaneously lose HBsAg and develop anti-HBs antibodies. Loss of HBsAg with or without emergence of anti-HBs (defined “functional” cure) is the goal of current treatment efforts, because it reduces the risk of liver cirrhosis, cancer, and liver-related death (9). Because of the unique life cycle of HBV, functional cure requires silencing or elimination of both cccDNA and integrated HBV DNA (9). While neither can be readily achieved by a finite treatment course with the currently available reverse transcription inhibitors, there is evidence that immune responses can be harnessed to contribute to functional cure. HBV, while not inducing innate cytokines such as type I IFN, TNF-α, and IL-6, is sensitive to them (10). This sensitivity explains why HBV/HCV-coinfected patients, in whom HCV induces an IFN-mediated activation of IFN-stimulated genes and natural killer cells, experience HBV reactivation when the IFN response is diminished after HCV is cleared (11). In vitro, high doses of IFN-α have been shown to affect different stages of the HBV life cycle, including the induction of epigenetic changes in cccDNA-bound histones (12), inhibition of HBV replication and transcription, and even degradation of cccDNA (reviewed in ref. 3). Therapy with pegylated IFN-α activates intrahepatic innate immune responses via induction of IFN-stimulated genes and stimulation natural killer cells (13), yet it results in HBsAg clearance in only a small percentage of patients.

With current efforts aiming at developing and combining novel treatments that target HBV replication, cccDNA, and HBsAg levels and/or to reinvigorate or substitute via adoptive transfer immune responses (14), there are still important virological and immunological aspects of chronic HBV infection that are not yet sufficiently understood. Translational research in well-defined, ideally prospectively followed patient cohorts may help to obtain insights into immune control that can be exploited to prevent the severe long-term effects of chronic HBV infection and to identify biomarkers for immune monitoring in the course of natural infection and during novel treatments. The following are some of the open questions:

*How does HBV establish chronic infection in early life?* Several studies in mice have identified potential factors that may contribute to the age-dependent differences in immune responses against HBV. While mice are not permissive for HBV due to the absence of the HBV entry receptor, it is possible to launch HBV replication from a transgene or an adenoviral vector. Adoptive transfer of naive immune cells into these mice during “adulthood” results in T and B cell priming and seroconversion from HBsAg^+^ to anti-HBs^+^ whereas adoptive transfer of immune cells into young transgenic mice does not (15). Factors that may contribute to this differential response include age-dependent expression of costimulatory molecules on antigen-presenting cells in the liver, decreased expression of chemokines that are required for optimal induction of virus-specific B cells, reduced T follicular helper responses, and age-dependent changes in gut microbiota (reviewed in ref. 16). Early exposure to secreted HBeAg during vertical transmission has also been implicated in this response (17). However, to date, there are almost no translational studies in humans. A notable exception is the demonstration of enhanced innate immune cell maturation and Th1 cell differentiation in cord blood from neonates of HBV-infected and uninfected mothers (18). This immunological state was associated with an enhanced ability of cord blood immune cells to respond in in vitro assays and may represent a state of trained immunity (18), consistent with the idea that HBV may be a symbiont that confers advantages to the host.

*Which immunological mechanisms prevent immune activation despite high levels of HBV DNA in the first decades after infection?* An important phase is the HBeAg^+^ noninflammatory phase of chronic HBV infection because HBV DNA integration and clonal hepatocyte expansion have been shown to start at this early stage (19) and shown to increase the risk of developing hepatocellular carcinoma (20). Patients at this stage have exceedingly high HBV DNA levels yet no/minimal evidence of liver inflammation. Even after cessation of antiviral therapy, this group does not experience the disease flares that are common in other patients with HBV. Intriguingly, functional HBV-specific T cell responses are detected in the blood of these patients (21), raising the question of how the immune response is regulated at this stage.

*Which factors drive the progression through the different phases of chronic HBV infection?* Host, viral, and environmental factors that drive the transition from noninflammatory to inflammatory phases and from HBeAg^+^ to anti-HBe^+^ status are still poorly understood. Due to the unpredictable timing of the transition between disease phases, there is a lack of prospective, immunological studies. Cross-sectional studies identified a decrease and ultimate loss of HBs-specific T cell responses with increasing age (22), a heterogeneous and complex profile of HBV-specific CD4^+^ and CD8^+^ T cells with increased expression of exhaustion markers (reviewed in ref. 11), altered energy metabolism, and mitochondrial dysfunction (23). However, the transcriptional profile of HBV-specific T cells is distinct from that of exhausted T cells in other viral infections (24), opening avenues for immunotherapy and restoration of T cell function. At the same time, studies on HBcAg and HBsAg-specific B cells have just recently started. Beyond antibody production, B cells can serve as antigen-presenting cells and exert regulatory function (Breg), and a dysfunctional phenotype has been reported (25). As such, there is a continuing need to perform studies on liver tissue to delineate the differential roles of HBV-specific immune cells and nonspecific, secondarily recruited bystander cells, the contribution of tissue-resident immune cells to HBV control versus disease, and the interactions of immune cells with each other and with parenchymal cells in the liver during the course of infection and novel interventions. New techniques, such as spatial transcriptomics and single-cell analysis will aid in this effort.

## Conclusions

Although our understanding of protective immunity against HBV has resulted in vaccination programs that significantly reduce infections, chronic liver disease, and hepatocellular carcinoma in countries that implemented them, there is still no cure for those who are currently chronically infected (estimated as 254 million people worldwide in 2022). Antiviral therapy does not eliminate cccDNA, and a multipronged approach that includes immune activation is likely necessary. A better understanding of virus-host interaction during the course of chronic HBV infection and therapeutic interventions is still needed to develop strategies to prevent the transition from noninflammatory to inflammatory phases of persistent infection, to induce immune control, and, ultimately, and to achieve functional cure.

## Figures and Tables

**Figure 1:**
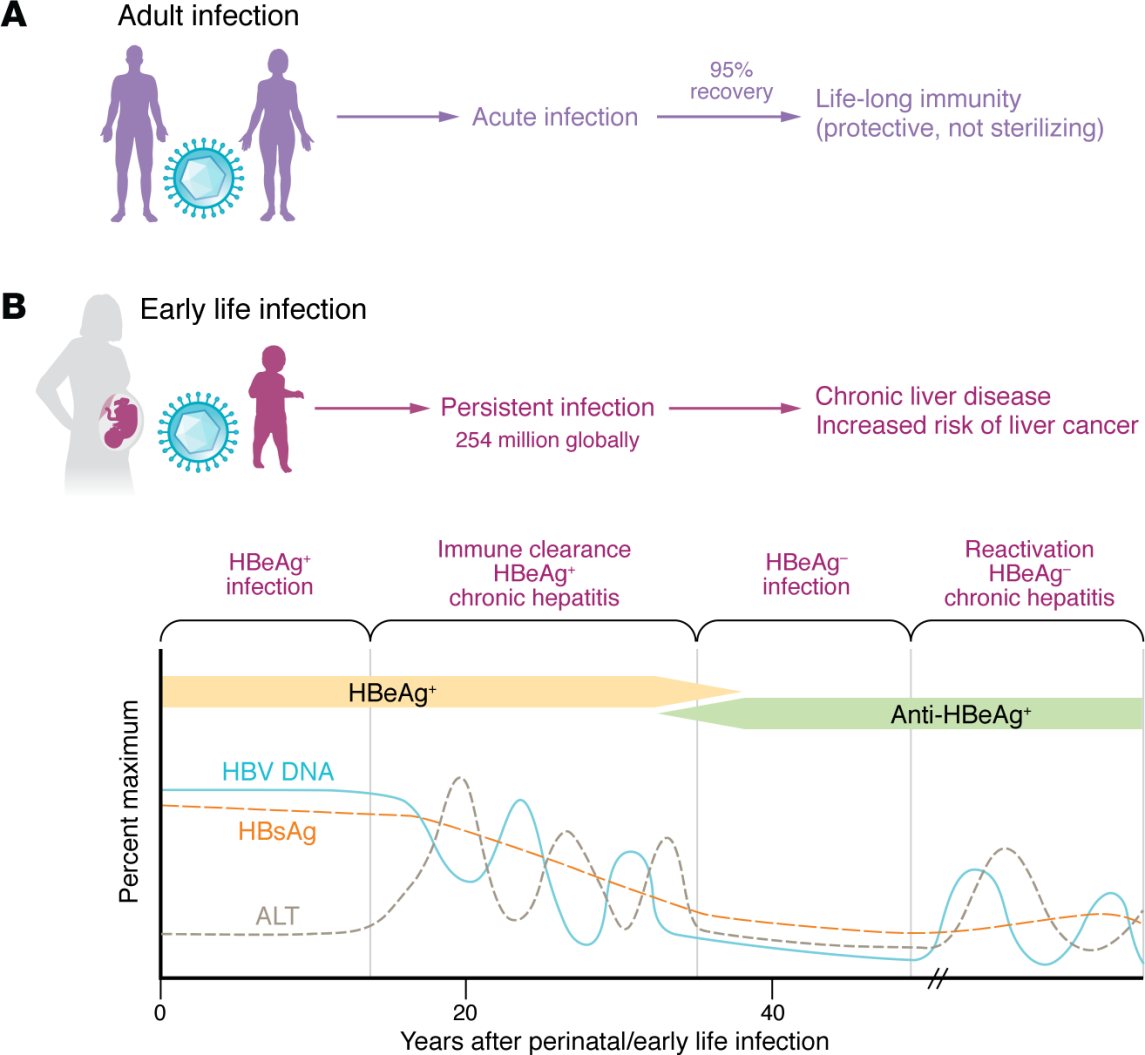
Course of acute and chronic HBV infection. (**A**) HBV infection during adulthood results in an acute, often severe phase of liver inflammation that is self-limited in the vast majority (95%) of cases and results in life-long cellular and humoral immunity. Immunity is protective but not sterilizing, as HBV can reactivate from cccDNA if the patients become immunosuppressed. (**B**) HBV infection in early life results in persistent, life-long infection, currently affecting more than 254 million people worldwide. Phases with normal alanine aminotransferase levels (HBeAg^+^ or HBeAg^–^ infection) alternate with phases of increased alanine aminotransferase levels (HBeAg^+^ or HBeAg^–^ hepatitis). Whereas both the acute and the chronic forms of HBV infection can be prevented by prophylactic HBsAg vaccination, there is no curative treatment for most patients once they acquired chronic HBV infection. Current treatment with nucleos(t)ide analogs suppresses HBV replication and reduces the risk of liver fibrosis and carcinoma but does not eliminate HBV cccDNA, and treatment with pegylated IFN-α rarely results in HBsAg loss. Of note, HBsAg can be produced from both cccDNA and from integrated, nonreplicating HBV DNA sequences. The latter are thought to be the dominant source of serum HBsAg in HBeAg^–^ patients. Loss of HBsAg with or without detectable anti-HBs is called functional HBV cure, if it persists for more than 24 weeks without concomitant therapy. Adapted from *Gastroenterology* with permission (16).

## Version 1 10/01/2024

Electronic publication
